# Urinary complement biomarkers in immune-mediated kidney diseases

**DOI:** 10.3389/fimmu.2024.1357869

**Published:** 2024-06-03

**Authors:** Vartika Kesarwani, Muhammad Hamza Bukhari, J. Michelle Kahlenberg, Shudan Wang

**Affiliations:** ^1^ Division of Rheumatology, Department of Medicine, Emory University School of Medicine, Atlanta, GA, United States; ^2^ Department of Medicine, Johns Hopkins Howard County Medical Center, Columbia, MD, United States; ^3^ Division of Rheumatology, Department of Medicine, University of Michigan, Columbia, MI, United States; ^4^ Division of Rheumatology, Department of Medicine, Montefiore Medical Center / Albert Einstein College of Medicine, Bronx, NY, United States

**Keywords:** complement, kidney disease, urine biomarker, lupus nephritis (LN), complement drug, primary membranous nephropathy, IgA nephropathy, C3 glomerulopathy

## Abstract

The complement system, an important part of the innate system, is known to play a central role in many immune mediated kidney diseases. All parts of the complement system including the classical, alternative, and mannose-binding lectin pathways have been implicated in complement-mediated kidney injury. Although complement components are thought to be mainly synthesized in the liver and activated in the circulation, emerging data suggest that complement is synthesized and activated inside the kidney leading to direct injury. Urinary complement biomarkers are likely a better reflection of inflammation within the kidneys as compared to traditional serum complement biomarkers which may be influenced by systemic inflammation. In addition, urinary complement biomarkers have the advantage of being non-invasive and easily accessible. With the rise of therapies targeting the complement pathways, there is a critical need to better understand the role of complement in kidney diseases and to develop reliable and non-invasive biomarkers to assess disease activity, predict treatment response and guide therapeutic interventions. In this review, we summarized the current knowledge on urinary complement biomarkers of kidney diseases due to immune complex deposition (lupus nephritis, primary membranous nephropathy, IgA nephropathy) and due to activation of the alternative pathway (C3 glomerulopathy, thrombotic microangiography, ANCA-associated vasculitis). We also address the limitations of current research and propose future directions for the discovery of urinary complement biomarkers.

## 1 Introduction

The complement system plays a central role in the pathogenesis of many immune-mediated kidney diseases (1–4). All parts of the complement system, including dysfunction of the complement regulator proteins, have been implicated in complement mediated kidney injury (5). The complement system is activated through three pathways: the classical, alternative, and mannose-binding lectin pathways, and is tightly regulated by regulator proteins (6). These three pathways converge to form the membrane attack complex (MAC, C5b-9), which lyses pathogens (7) and induces sub lytic proinflammatory intracellular signaling in eukaryotes (8) in a variety of autoimmune settings such as lupus nephritis and anti-neutrophil cytoplasmic antibodies (ANCA) associated vasculitis.

Complement deposition in kidney tissue has been found in various glomerulonephritides, including lupus nephritis, membranous nephropathy, IgA nephropathy, C3 glomerulopathies, ANCA-associated vasculitis, and thrombotic microangiopathies (3, 9–11). Beyond immune complex deposition, emerging data shows that complement is being synthesized inside the kidney and can cause direct injury through various mechanisms such as 1) cytokine production contributing to interstitial inflammation (12), 2) direct stimulation of extracellular matrix production (12–15) and 3) activation of the renal renin angiotensin system leading to progressive kidney injury and damage (16).

With the rise of therapies targeting the complement system, there is a critical need to better understand the role of complement in kidney disease. Additionally, there is a need to discover dependable complement biomarkers that would be instrumental in evaluating disease severity and guide personalized treatments. Most studies of complement association with kidney diseases involve biomarkers of complement activation in serum or plasma and their deposition on the tissue surface, as seen on kidney tissue biopsy. However, the reliability of serum complement monitoring may be challenging as it can be affected by systemic inflammation. It is thought that complement-derived peptides in urine may be a better assessment of intrarenal complement activation (17, 18). For example, urine complement activation products are undetectable in healthy individuals (18), but significantly increased in patients with immune mediated kidney diseases such as lupus nephritis and IgA nephropathy (19). Urinary complement excretion correlated with complement deposition in kidney tissue, disease activity, proteinuria, creatinine and worse kidney outcomes in multiple studies (18, 20, 21). Therefore, it has been proposed that monitoring the dynamic changes in urinary complement proteins, rather than measuring serum complement levels, may better reflect the disease activity and pathogenic mechanisms and help guide therapeutic interventions. In addition, urinary biomarkers have the advantage of being non-invasive and easily accessible, as compared to complement immunohistochemistry staining on kidney tissue, which will require a kidney biopsy.

The overall purpose of this review is to assess the available evidence regarding the role of urinary complement biomarkers in monitoring disease activity, determining prognosis, and assessing treatment response in patients with immune-mediated kidney diseases. We will also discuss the limitations of these urinary biomarkers and the future for developing commercial assays for these diseases.

## 2 The pathways of complement activation

The complement system is an essential part of the innate immune system, which is composed of over 20 proteins that work together to destroy pathogenic organisms, activate and control the adaptive immune response. The complement system can be activated through three different pathways: (a) the classical pathway or “antibody-dependent activation,” (b) the alternative pathway or the “antibody-independent activation,” and (c) the mannose-binding lectin (MBL) pathway (6).

**Figure 1:**
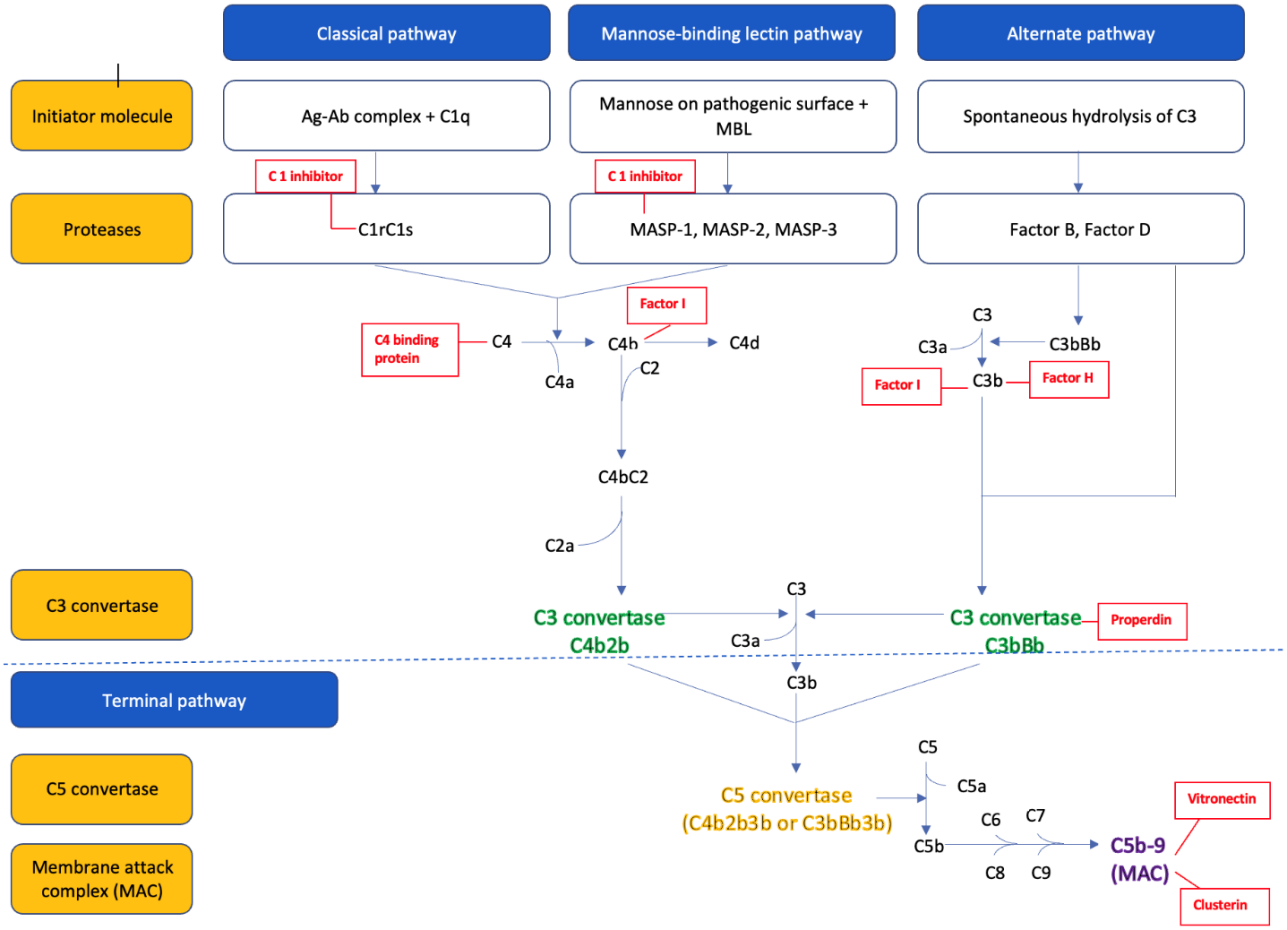
The pathways of complement activation and plasma derived complement regulators (in red).

The parts of the three complement pathways are summarized in **Figure 1** . In brief, the ***classical pathway*** is initiated by C1q binding to the Fc portion of the IgM and IgG antibodies (antigen-antibody complex), which activates the serine proteases C1r and C1s. The activated C1s then cleaves the C4 into C4a and C4b, and C2 into C2a and C2b. The C4b and C2b fragments combine to form the C3 convertase (C4bC2b) of the classical pathway (22).

The ***alternative pathway*** is constantly activated in the body by spontaneous hydrolysis of the C3 into C3a and C3b. C3b combines to factor B, to form C3bB. Factor B is then cleaved by factor D to yield the C3 convertase (C3bBb) of the alternative pathway. This C3 convertase is stabilized by the binding oligomers of factor P (properdin). The C3b generated from the above process further binds to the pathogenic cell surface to form more C3 convertase and therefore leads to the ‘amplification’ of the alternative pathway (6, 22).

The ***mannose-binding lectin (MBL) pathway*** is activated by the binding of MBL protein to the mannose residue found on the surface of many common pathogens. This in turn activates the MBL associated serine proteases (MASP), MASP-1 and MASP-2, which then cleaves C4 into C4a and C4b, and C2 to C2a and C2b. The C4b and C2b combine to form the C3 convertase (C4b2b) of the MBL pathway (23).

All three pathways converge to a ***common pathway*** that leads to cleavage of the inactive central component C3 to biologically active C3b and C3a fragments via the C3 convertase. The C3 convertase can then bind to another molecule of C3b to form the C5 convertase, which cleaves C5 into C5a and C5b. C5b combines with other complement proteins (C6, C7, C8, and C9) to form a membrane attack complex (MAC, C5b-9), part of the terminal complement pathway. The MAC attaches to the pathogen’s surface to form a transmembrane channel and leads to the osmotic lysis of the target cell in pathogens and induces sub lytic proinflammatory intracellular signaling in eukaryotes in a variety of autoimmune settings such as in lupus nephritis (7).

C5a and C3a formed during this process serve as potent chemo attractants that recruit other inflammatory cells such as neutrophils, eosinophils, monocytes, and T-lymphocytes to the site of injury. Other smaller fragments generated by complement lysis such as inactive C3b (iC3b), C3dg, and C3d serve as “opsonin” that tag the organism for destruction by other immune cells.

As uncontrolled activation of the complement system can damage the host tissue, it is tightly controlled by many ***regulatory proteins*** (24). These regulatory proteins, either circulating in plasma or attached to cell membranes, regulate the complement pathways at several critical stages: activation, amplification, and MAC formation. This regulation is achieved by preventing the formation of convertases, facilitating their rapid dissociation, and mediating the proteolysis of activation derived fragments. For instance, C1q, a plasma regulator protein, inhibits activation of the classical and MBL pathways by neutralizing the C1s/C1r and MASP complex. Additionally, several regulatory proteins, plasma-derived (C4 binding protein, factor H, factor I, properdin) and membrane-bound regulatory proteins (CD55, CD46, CD35, CSDM1) prevent the amplification of the complement pathway by preventing the formation of and promoting disassembly of the C3 and C5 convertases. Regulation also extends to the formation of the MAC; plasma proteins like, protein S or vitronectin and clusterin bind to MAC complex inhibiting its attachment to cell membranes. Conversely, CD59, a membrane-bound protein inhibits the final steps of MAC assembly on host tissues (25).

The imbalance between the complement activators and regulators is increasingly recognized as the pathogenic mechanism for many kidney diseases such as lupus nephritis, IgA nephropathy, atypical hemolytic uremic syndrome (aHUS), C3 glomerulopathy, and antineutrophil cytoplasmic antibody (ANCA) associated vasculitis, and among others which will be discussed in this review (26, 27).

## 3 Proposed mechanisms of complement-mediated kidney injury

**Figure 2:**
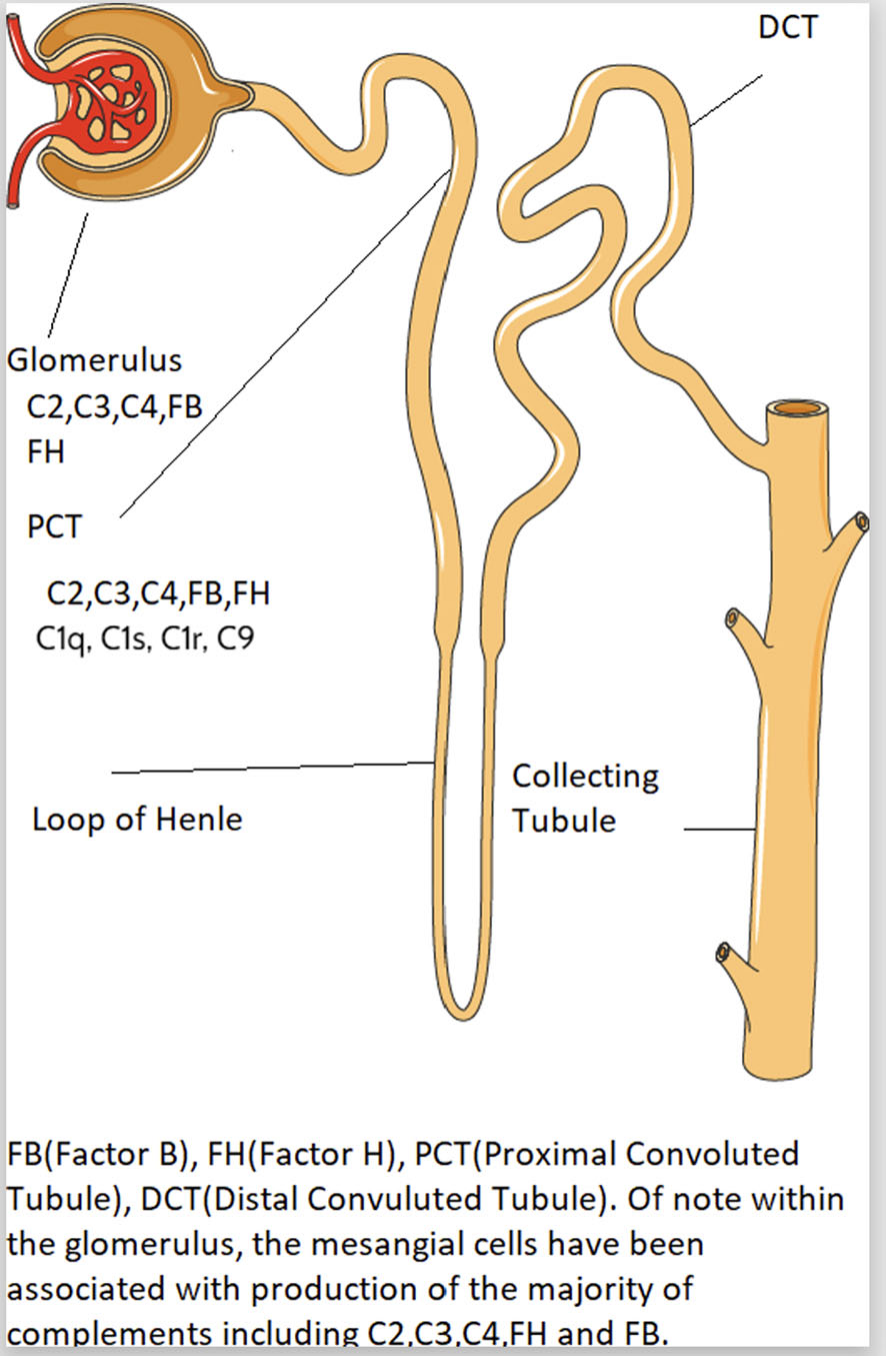
The endogenous synthesis of complement proteins by the kidney.

Intrarenal complement production has been implicated in the pathogenesis of immune mediated kidney diseases. Although complement components are thought to be mainly produced in the liver and activated in the circulation, emerging data suggest complement is synthesized and activated inside the kidney and can lead to direct kidney injury (5, 28). The location of intrarenal complement synthesis is summarized in **Figure 2**. Complement activation can lead to tissue injury through 1) generation of chemoattractant (C3a and C5a) which lead to vascular permeability and leukocyte infiltration and 2) formation of the cytotoxic MAC that act to lyse cells and propagate inflammatory pathways (29). **Table 1** summarizes the various pathways implicated in various immune-mediated kidney diseases.

**Table 1:** Complement pathways implicated in various kidney diseases.

Disease	Classical pathway	Alternative pathway	Lectin pathway	Complement regulator proteins
Immune-mediated kidney diseases				
**Lupus nephritis**	✔	✔	✔	✔
**IgA nephropathy**	–	✔	✔	✔
**Primary membranous nephropathy**	–	✔	✔	✔
**C3 glomerulonephritis**	–	✔	–	✔
**ANCA-associated vasculitis**	–	✔	–	–
**Thrombotic microangiopathies**	–	✔	–	✔

✔, involvement of the complement pathways in the disease specified.

There is a link between complement gene expression and tissue injury. Prior studies showed that the intrinsic kidney tubular epithelial cells and interstitial kidney cells express high level of complement genes such as C1q, C1r, C1s and C3 (5), which could cause kidney damage through the following potential mechanisms: 1) cytokine production contributing to interstitial inflammation (12), 2) direct stimulation of extracellular matrix production (12–15) and 3) activation of the renal renin angiotensin system (16). For example, Xavier et al. (2017) reported local synthesis of intracellular C1q by mouse kidney interstitial cells, leading to increased inflammation and kidney scarring via increased production of IL-6, monocyte chemoattractant protein-1 and macrophage inflammatory protein-1 (MIP1) alpha (13). However, other studies showed early complement component C1q function to clear apoptotic cells and immune complexes, where C1q-deficient individuals develop early onset of SLE (30).

To determine the role of C1 complex proteases C1r and C1s on kidney fibrosis, Xavier et al. (2019) found increased expression of both mRNA and protein levels of C1r and C1s in kidney tubular epithelial cells in mice with kidney fibrosis using both immunohistochemistry and *in situ* hybridization studies. In addition, mice with C1r deletion had reduced complement component C3, decreased acute inflammatory responses and reduced proliferation of connective tissue cells (e.g., platelet-derived growth factor receptor-β) during folic acid mediated tubulointerstitial fibrosis, as compared with mice with intact C1r (12). Another study showed C3 deposition in kidney tubules was found in mice with tubulointerstitial fibrosis. Inversely, C3 deficient mice had reduced C3 deposition in kidney tissues and reduced macrophage infiltration in kidney cells (12). These studies suggest activation of C3 through all three pathways may play a role in kidney injury.

The mannose binding lectin pathway has also been proposed to be involved in kidney injury. Studies showed that mice with MASP-2 and collectin 11 (CL11) deficiency, both key components of the lectin pathways, had less evidence of complement activation and kidney injury (31). CL11 was thought to propagate tubulointerstitial fibrosis through kidney fibroblast proliferation and leukocyte chemotaxis (32).

The formation of the MAC through the activation of the terminal pathway has been implicated in tubulointerstitial kidney injury based on animal nephrogenic models. Prior studies showed that complement sufficient rats had evidence of MAC deposition in the proximal kidney tubules, increased tubulointerstitial inflammation, and fibrosis (as evident by vimentin and osteopontin staining) as compared to C6-deficient rats (33, 34). However, there was no difference in C3 deposition between C6 sufficient and deficient rats, thereby emphasizing the role of the MAC in mediating tubular kidney damage in proteinuric kidney models (35, 36). Another study demonstrated that C6-deficient mice with proteinuric urine (which is nephrotoxic) had reduced progression of tubular injury and renal failure beyond a 35–70-day period. This suggests that complement activation may mediate long-term progressive tubulointerstitial injury beyond the initial acute stressors of kidney injury attributable to increased flow and angiotensin-2 activation (37). In our study of 30 lupus nephritis (LN) patients, we showed that MAC deposition in the kidney tubules was associated with interstitial fibrosis (IFTA), the histological equivalent of chronic, irreversible kidney damage (38). In addition, glomerular MAC deposition was associated with hypertension, male gender and poor response to standard lupus nephritis treatment (39).

Membrane-bound and soluble complement regulators have a protective role in controlling complement mediated inflammation. An imbalance of complement activation versus regulators may contribute to disease state. Laskowsky et al. (2016) found that a lack of expression of Factor H and complement receptor 1- related protein y (Crry) was associated with increased kidney inflammation and C3 deposition in kidney cells (40).

Bao et al. (2011) transplanted kidneys from Crry and C3 negative mice into hosts lacking C3a and/or C5a receptors. These authors found reduced tubulointerstitial inflammation and fibrosis in the C3a receptor-deficient mice (41). Peng et al. (2012) also demonstrated reduced ischemia-reperfusion injury in murine models lacking these receptors (42). Similarly, in lupus nephritis models, studies show increased survival and improved kidney disease with blockage of C5 and C5a receptors. Therefore, complement receptors also have a role in pathogenesis of kidney injury.

Elucidating the mechanisms of complement-mediated kidney injury is especially relevant given multiple complement targeting therapies are in the pipeline for a wide variety of kidney diseases including but not limited to lupus nephritis, IgA nephropathy, and ANCA associated vasculitis (29, 43). The complement drugs are targeting different parts of the complement pathways such as C5 inhibitors eculizumab and ravulizumab, C3 inhibitor (pegcetacoplan APL-2), C1 inhibitors, MASP 1 and MASP2 inhibitors, C5a inhibitors, and Complement Factor D inhibitors for a wide range of kidney diseases (44). Given the approval of iptacopan (inhibitor for Factor B) for paroxysmal nocturnal hemoglobinuria and its current investigation for several complement mediated kidney diseases, Factor B may also be an emerging potential target protein for future therapies involving complement inhibitors.

## 4 The rationale and evidence for urinary complement markers

Traditionally, complement deposition in kidney tissues has been shown to correlate with histological severity and disease activity (38, 39, 45). In addition, serum or plasma levels of complement activation products have been used to assess complement activation and disease activity. However, these serum and/or plasma complement products are neither sensitive nor specific in assessing disease activity and predicting treatment response as their levels can be confounded by systemic inflammation (46). Given the role of intrarenal complement activation, urinary complement products may better reflect disease activity.

Using lupus nephritis as an example, the current standard of practice for detection and monitoring of lupus nephritis involves the measurement of laboratory parameters such as proteinuria, urine protein: creatinine ratio, creatinine clearance, serum complement levels (C3, C4), and serum anti-dsDNA antibody levels. These markers are neither sensitive nor specific for lupus nephritis for various reasons. Firstly, they fail to differentiate between kidney disease activity (glomerular complement activation), which occurs earlier in the disease course from chronic kidney damage (47). Secondly, these markers are confounded by the preexisting chronic systemic inflammation in systemic lupus erythematosus (SLE) and therefore do not accurately reflect disease activity (48). The superiority of urinary complement biomarkers compared to conventional serum biomarkers was demonstrated in a study by Manzi et al. In this study of 31 patients with SLE, serum and urine complement levels were measured at 3 consecutive visits, 4 months apart. The authors found that complement split products C4d and Bb were more sensitive predictors of future moderate to severe disease activity than serum C3 and C4 (sensitivity 65 to 93% as compared to 53 to 64%), However, urinary C3d levels best correlated with kidney disease activity and were superior to all the plasma markers C3, C4d, Bb, C5b-9 and anti-dsDNA antibody levels in distinguishing patients with active lupus nephritis from those without (P-value =0.02) (49).

Kidney biopsy remains the gold standard for establishing the diagnosis, prognosis and for guidance of therapy in lupus nephritis (50). However, it is an invasive procedure and cannot be conducted serially. It has been proposed that urinary biomarkers reflect the real-time status of kidney inflammation and could therefore serve as ideal biomarkers of lupus nephritis. Urinary complement biomarkers have the advantage of being non-invasive and easily accessible. These biomarkers may delay or prevent the need for a kidney biopsy to evaluate disease progression in in various kidney diseases (51). More importantly, with the emergence of complement inhibitors, there is an urgent need for complement biomarkers to identify non-responders with excess complement activation and who may benefit from novel complement targeting therapies as part of precision medicine (44).

## 5 Methods used to assess complement proteins

For blood, urine and biopsy samples collected for analysis, ELISA or mass spectrometry have been the primary methods used to analyze complement proteins. Regarding sample collection, Brandwijk et al. did a literature review and noted that to assess individual complement markers in plasma, an EDTA tube should be used, processed within 1 hour and put on ice immediately. Sample should be centrifuged for 10 min at 2000xg at 4 degrees Celsius and then stored at -80 degrees Celsiusspectrometry (52).

Zhang et al. illustrated various ELISA kits available to detect urinary and plasma complement activation products in primary membranous nephropathy. They used kits by Quidel to detect MBL, C4d, Bb, properdin, C3a, C5a and soluble C5b-9 in plasma and urine samples. They followed a 5-step process which involved micro assay plates being pre coated with monoclonal antibodies specific to the complement being evaluated. The samples from urine and plasma were diluted and incubated at room temperature per the kit manufacturer’s instructions and subsequently horseradish peroxidase conjugated antibodies were added and bound to the complement components. Using the Biorad 550 kit, the results were recorded as net optical absorbance and the results were corrected by urine creatinine concentrations (53).

Mass spectrometry is another method for detecting complement products in urine, plasma, or biopsy samples. As in ELISA, different forms of commercially available kits and methods are used to analyze samples using mass spectrometry. As an example, our group detected over 18 urinary complement proteins using mass spectrometry. Briefly, approximately 500 microliters of urine were processed and centrifuged immediately after thawing for each sample. Buffer exchange was performed using spin filters with a 10 kDa molecular cutoff, and proteins were digested directly on the filter. Peptides eluted from the filters were separated using nano-liquid chromatography – for enhanced sensitivity – coupled online with high-resolution tandem mass spectrometry (Orbitrap Fusion Lumos, Thermo Scientific). All proteins detected were normalized by urine creatinine excretion to account for differences in concentration (19).

Sethi et al. analyzed kidney biopsy samples for complements and downstream protein analysis in different forms of glomerulonephritis. They used laser dissection of kidney biopsy samples to identify proteins in formalin-fixed-paraffin-embedded specimens. Proteins were then denatured and digested with a commercially available trypsin (Promega) and the resultant peptide was run through a QExactive-Plus mass spectrometer (Thermo-Fisher) coupled to a nano flow high performance liquid chromatography system. Resulting spectra were then analyzed using Mascot and X!Tandem (54).

## 6 Kidney diseases due to immune complex deposition

### 6.1 Lupus nephritis

Complement activation plays an essential role in the pathogenesis of lupus nephritis. The deposition of circulating immune complexes in the kidneys and subsequent activation of the classical complement pathway was considered the predominant mechanism mediating tissue injury in lupus nephritis (55). However, recent studies have demonstrated that the alternative pathway activation in lupus nephritis is also associated with worse kidney outcomes and poor treatment response compared to patients with glomerular classical pathway activation alone (55, 56). The role of the MBL pathway in the pathogenesis of lupus nephritis is not fully understood (57).

Given the known role of the classical complement pathway activation in lupus nephritis, some have investigated urinary markers C3d, a degradation product of C3, as a potential marker of classical pathway activation and lupus nephritis disease activity (58, 59). Negi et al., compared serum and urinary levels of C3d among 4 groups of patients (controls, SLE with inactive disease, SLE with active non-renal disease, SLE with active renal disease), and found that urinary C3d was better than serum C3d in differentiating active vs. nonactive SLE. Serum C3d was elevated in both active and nonactive SLE, while urine C3d was only elevated in active SLE with kidney involvement. Urinary C3d has a sensitivity of 100% and specificity of 88% in differentiation of active renal from extra renal SLE, which is better than the sensitivity of existing biomarkers of low serum C3 and high dsDNA (sensitivity of 63% and 88% respectively) (60). Another study by Kelly et al., compared the correlation of urinary C3d levels with proteinuria and found that urinary excretion of C3 fragment correlated with proteinuria among 28 SLE patients. However, this study was not conclusive because C3 fragments were also present among SLE patients with non-renal manifestations (61).

To evaluate the change in urinary C3 and C4 degradation products (C3d and C4d) over time as they relate to treatment response, Ganguly et al. measured urinary C3d and C4d levels among 28 patients with biopsy-proven active lupus nephritis, 4 patients with inactive lupus nephritis, and 10 healthy controls at baseline and at 3-month follow-up after lupus nephritis treatment (majority received cyclophosphamide) and assessed its correlation with disease activity scores (SLE disease activity index [SLEDAI] 2K, renal SLEDAI) and urinary protein/creatinine ratio (UPCR). The authors found urinary C3d/creatinine correlated with disease activity markers SLEDAI 2K, renal SLEDAI, and UPCR at baseline. A urinary C3d/creatinine cutoff of 67.39 ng/mg was found to have a sensitivity of 100% and a specificity of 75% in differentiating active from inactive lupus nephritis. They also found that responders had a bigger decrease in urinary C3d/creatinine levels 3 months post treatment, as compared to non-responders who had persistently elevated urinary C3d levels. However, this study did not find C4d to correlate with disease activity or treatment response. Therefore, this study concluded that a fall in urinary C3d levels at 3 months can be used as a marker of treatment response and reinforced the importance of change in urinary complement levels over time instead of absolute value alone. Such patients can be assessed for early change in treatment to prevent irreversible renal damage (58).

C4 has also been proposed as a marker of disease activity. In a study by Ueda et al, urinary C4 was greater in SLE patients with proteinuria (>1 g/day) compared to healthy controls without SLE. Urinary C4 showed no correlation with traditional disease activity markers (serum C4, anti-dsDNA) but correlated with proteinuria. On serial measurement, urinary C4 decreased in parallel with increase in serum C4 and anti-ds-DNA in response to treatment in 11 out of 13 patients receiving high doses of steroids. In these patients, urinary C4 decreased in a month after treatment while the decrease in total urinary protein occurred afterward, suggesting that improvement in urinary C4 precedes improvement in proteinuria. In 2 out of 13 patients, urinary C4 levels increased and correlated with disease flare-up in one patient and preceded the flare-up in other, indicating that urinary C4 may predict disease exacerbations. This study was limited by small sample size (62).

In addition to looking at C3d, C4d and C4, other studies have evaluated the terminal complement pathway as a potential marker of disease activity and kidney damage. In our study of 46 biopsy proven lupus nephritis patients, we compared the urinary complement profiles between patients with and without interstitial fibrosis/tubular atrophy (IFTA). We found that lupus nephritis patients with moderate to severe IFTA have increased urinary C3, CFI, and C9-to-CD59 ratio as compared to those with none to mild IFTA (19). This study highlights the importance of looking at the balance between complement activation (e.g., MAC complex) and regulation (e.g. CD 59) in evaluating complement biomarkers and suggests C3, CFI and C9-to-CD59 ratio may be a marker of tubulointerstitial disease in lupus nephritis.

Li et al. looked at MAC activation and histological disease activity on kidney biopsy. They found that highest urinary complement activation products (CAPs) excretion, namely C5a, C5b-9, and factor Bb, was found in proliferative and proliferative + membranous nephropathy as compared to lupus nephritis class I, II or V among 149 SLE patients undergoing kidney biopsy for suspected lupus nephritis. All three CAPs correlated with an increased Activity Index, but the strongest association was seen with C5b-9. Only a modest correlation was present between Chronicity Index and urinary Bb and C5a excretion. The study thus concluded that urinary C5b-9 reflects histological activity and could serve as a potential biomarker of treatment response with anti-complement therapies in lupus nephritis (63). Similarly, Schulze et al. showed increased urinary excretion of MAC and C5, with a modest correlation with urinary protein excretion among 18 LN patients; however, its correlation with disease activity and treatment response was not assessed in this study (64). Another study by Tamano et al. that included 104 lupus nephritis patients demonstrated increased urinary excretion of complement factor H (CFH), a regulator of the alternative complement pathway, in all patients with LN (65) but the authors did not further investigate the correlation of urinary CFH excretion with disease activity or treatment response.

To evaluate multiple complement proteins at once, Zhao et al. compared urinary complement proteins between 24 LN patients with active SLE (SLEDAI 2K >5) and low activity SLE (SLEDAI 2K <5). They found differentiating levels of 14 complement pathway proteins – 8 were upregulated (C3, C4b, C5, C7, C8 alpha subunit, C8 gamma subunit, vitronectin, CFH) and 6 were downregulated (mannose-binding lectin serine protease-2, C6, CFD, and VSIG-4) in active SLE. Of these, urine C9, C8 alpha subunit, C4b, and C8 gamma subunit were significantly positively correlated with SLEDAI-2K and were highly discriminatory in determining overall SLE disease activity. The reported sensitivity of C9 and C8 alpha subunit was 92% and specificity of 50–75%. The sensitivity of C4b and C8 gamma subunit was ~50% and specificity was 91–100%. This study did not evaluate the correlation of these urine markers with renal disease activity in lupus nephritis (66).

Urinary complement biomarkers, particularly urinary C3d, C4, CFI and C5b-9 have shown promise as reliable tests to identify active lupus nephritis, monitor disease activity, assess treatment response, and predict disease flare in a few studies. However, these markers have not been widely accepted in clinical practice given the lack of substantial supporting data. Therefore, there is currently no widely accepted consensus regarding the role of urinary complement biomarkers in management of lupus nephritis. Further validation studies in large population cohorts are needed to investigate and establish their role as potential biomarkers in SLE.

### 6.2 IgA nephropathy

IgA nephropathy is characterized by mesangial IgA deposits linked with the deposition of complement components, which suggests that complement activation is an important pathogenic factor in IgA nephropathy (67). Activation of the alternative and mannose-binding lectin pathway has been implicated in the pathogenesis of IgA nephropathy. Hiemstra et al. (68) demonstrated through animal studies that IgA directly activates the alternative pathway. Similarly, Anja Roos et al. (69) studied 60 biopsy samples of human patients with confirmed IgA nephropathy and found that 15 subjects contained mannose binding lectin(MBL), and all of those subjects were found to have co deposition of L ficolin, MBL associated serine proteinases and C4d, indicating activation of the lectin pathway. Therefore, the above studies have shown that IgA directly activates the alternative pathway, while the activation of MBL may be linked to the binding of polymeric serum IgA to MBL (68, 69).

Various studies have shown that the involvement of the MBL pathway, as evidenced by glomerular C4d and MBL deposition, is associated with progressive disease and worse kidney outcomes (69, 70). Alteration in factor H, an important regulator of the alternative pathway, has also been associated with the development and progression of IgA nephropathy. Deleting complement factor H-related genes 1 and 3 (CFHR1/3) is protective against IgA nephropathy, while increased plasma levels of FHR-1/Factor H and FHR-5 (antagonists of FH) are associated with progressive kidney disease.

Since the local intrarenal complement activation, not systemic activation of the complement system is linked to the progression in IgA nephropathy, it has been proposed that assessment of complement activation markers in the urine may be a reliable indicator of local disease activity (70, 71). This was demonstrated in a recent study by Seggara-Medrano et al. that included 98 patients with IgA nephropathy and demonstrated that urinary excretion of C4d and MBL showed a good correlation with the glomerular C4d and MBL deposition (reported sensitivity of 84–90% and specificity of 73–82%) (72).

Unlike lupus nephritis, studies in IgA nephropathy included urinary complement markers of the lectin pathway given its role in this disease. A study by Wang et al. that included 100 IgA nephropathy patients with varying proportions of glomerular crescent formation demonstrated that urinary levels of C3a and C5a (representing the common pathway), C4d and MBL (representing MBL pathway), and C5b-9 (representing terminal pathway) were positively correlated with the proportion of glomerular crescents on renal biopsy, serum creatinine levels, and proteinuria. The correlation between glomerular crescent formation and urinary complement levels was strongest with urinary C4d excretion, highlighting the association of the lectin pathway with severe kidney disease. In this study, no association was found between the serum complement products and crescents or clinical parameters (73). The results of this study were in accordance with another study by Liu et al. that included 162 patients with biopsy-proven IgA nephropathy. The study demonstrated high urinary excretion of MBL in IgA nephropathy, which increased as histopathological phenotypes upgraded, and correlated significantly with the clinical predictors for the prognosis of IgA nephropathy (72).

It has also been demonstrated that glomerular deposition of alternative pathway regulators, factor H (fH), and properdin is associated with IgA nephropathy disease activity. A study by Onda et al. that included 71 patients demonstrated that the urinary excretion of MAC, Factor H, and properdin levels was higher in patients with IgA nephropathy, increased with disease severity, and showed a positive correlation with the percentage of glomerular sclerosis, serum creatinine, and proteinuria (74). These findings were also seen in another study by Zhang et al. that showed the association of high urinary levels of Factor H with the ratio of glomerular sclerosis, crescents, serum creatinine, and degree of proteinuria while studying 202 human patients with IgA nephropathy with renal biopsy as well as urine studies obtained (75). Another study by Liu et al. that included 351 patients with IgA nephropathy followed for ~51 months showed that high urinary levels of Factor H at the time of diagnosis were associated with worse kidney outcomes in the follow-up period (76). It is unclear if the complement regulators are playing a pathogenic role or serving as a bystander of overall increased complement activation in a diseased state.

### 6.3 Primary membranous nephropathy

Primary membranous nephropathy is an autoimmune glomerular disease that is one of the leading causes of nephrotic syndrome in adults. Most patients with primary membranous nephropathy have autoantibodies that bind antigens expressed by podocytes, in most cases anti-phospholipase A2 receptor (PLA2R) (70–90%) and thrombospondin type 1 domain containing 7A (THSD7A) (2–3%). These autoantibodies are predominantly of the IgG4 subclass, which cannot bind C1q and thus cannot activate the classical pathway (77). The IgG4 antibodies bind to and activate the MBL pathway leading to cytoskeletal alterations in human podocytes *in vitro*; the suggested pathogenic pathway (78, 79). Additionally, there is also evidence of alternative pathway activation in primary membranous nephropathy as supported by genetic evidence for the deposition of PLA2R antibody, C3, and C5b-9 in patients with MBL deficiency (80) and via production of antibodies targeting CFH (81).

While complement-related biomarkers have been associated with primary membranous nephropathy in many mouse model-studies, there is paucity of data regarding the role of urinary complement activation products as biomarkers of disease activity and treatment response in this group. A few studies have investigated the correlation of urinary complement activation products excretion with glomerular complement protein deposition and proteinuria thereby supporting the hypothesis that complement activations play a central role in the pathogenesis of primary membranous nephropathy. None of these studies evaluated the role of complement as a marker in predicting disease activity/flares and monitoring disease response.

A study by Ayub et al. suggests complement is activated inside the kidney and correlates with proteinuria. This study included 11 patients with biopsy-proven primary membranous nephropathy and measured the complement proteins in kidney biopsies and in the urine of these patients. The study found that eight proteins of the complement pathway (C1q, C3, C4, C5, C6, C7, C8, and C9) and 5 complement regulators (complement receptor type 1[CR1], factor H [FH], FH-related protein 2 [FHR2], vitronectin, and clusterin) were elevated in kidney biopsy samples of primary membranous nephropathy compared to healthy controls. The authors suggested that the presence of these complement depositions in glomeruli does not by itself indicate that intrarenal complement activation is occurring. In addition, they measured the urinary levels of complement activation products (CAPs) – Ba, C5a, and MAC which were all elevated in patients in primary membranous nephropathy compared to healthy controls and correlated with the degree of proteinuria (spot urine protein creatinine ratio), mainly in patients with heaviest proteinuria. The authors suggested that the presence of CAPs in the urine is suggestive of intra-renal complement activation because the molecular weight of MAC is around 1,000,000 Da, which is too large to be filtered, even in nephrotic patients. The intrarenal origin of uC5a is less certain (82).

Schulze et al. found similar results. They included 40 patients with biopsy-proven membranous nephropathy and measured the urinary excretion of MAC and C5. Random urine samples were obtained and showed that urinary MAC and C5 levels were significantly elevated in patients with membranous nephropathy as compared to proteinuric patients with biopsy proven non-membranous glomerulonephritis, and urinary MAC excretion showed a weak correlation to urinary protein excretion. This study was only aimed to support the pathogenesis of primary membranous nephropathy and not to explore the role of these complements as biomarkers for the disease (64).

Endo et al. included 20 patients with biopsy-proven primary membranous nephropathy investigated the role of factor H, a complement regulator protein in primary membranous nephropathy. They compared the complements seen on immunohistochemical staining of kidney biopsy samples with urinary excretion of CFH and C5b-9. The study reported intense glomerular staining C5b-9 and intense to moderate glomerular deposition of CFH with C3b and C3c. The authors found that the mean levels of urinary CFH were elevated in primary membranous nephropathy compared to health controls. There was no significant correlation between urinary excretion of factor H and urinary protein, GFR, and urinary C5b-9 level. The ratio of urinary C5b-9 to urinary factor H (UC5b-9/UfH) was closely related to GFR but not to urinary protein (83).

A study by Zhang et al. that included 134 patients with biopsy-proven primary membranous nephropathy and 25 healthy controls investigated the plasma and urinary complement levels in these two groups and studied its correlation with clinical data, treatment response, and kidney outcomes. The study found that urinary levels of C4d, MBL, C5a, C3a, and MAC were higher and urinary levels of C1q, Bb, and properdin were lower in primary membranous nephropathy compared to healthy controls. A similar pattern was seen in plasma complement levels, most remarkably elevated plasma C5a and C3a. MAC was only elevated in the urine and not the plasma, indicating *in-situ* renal complement activation. The urinary levels of C5a showed a positive correlation with urinary protein and PLA2R antibody levels in PLA2R positive patients; urinary MBL and C4d showed a positive correlation with urinary protein in both PLA2R positive and negative patients; and urinary Bb was positively correlated with urinary protein in PLA2R negative patients only. There was no inter-correlation between urinary and plasma complement levels. The study showed no correlation of plasma or urinary complements with treatment response and kidney outcomes (53).

In conclusion, many urinary excretions of CAPs, particularly those associated with the alternative and MBL pathway, and complement regulator proteins correlated with kidney deposition of these proteins in kidney biopsy samples and showed association with degree of proteinuria in most studies except one. This evidence indicates that urinary complement proteins can serve as potential biomarkers for disease activity but their role in predicting disease outcomes and treatment response is yet to be confirmed.

## 7 Kidney disease due to alternative complement pathway activation

### 7.1 C3 glomerulopathies

C3 glomerulopathy is a rare group of kidney diseases driven by complement dysregulation. It is characterized by the glomerular accumulation of C3 with little or no immunoglobulin deposition on kidney biopsy. Dysregulation of the alternative complement pathway in the fluid phase is the main pathogenic mechanism behind C3 glomerulopathies. This may occur either due to the development of autoantibodies or genetic alterations leading to dysfunction of the convertase (C3 and CFB gene) and the complement regulators (CFI, CFH, and CFHR5). The most frequently identified auto-antibody, C3 nephritic factor, targets and stabilizes the C3 convertase leading to the amplification of the complement cascade. Autoantibodies against C5 convertase (C5 nephritic factor), factor H, and factor B, although less frequent, are also seen. Genetic alterations are observed in ~25% of the patients with C3 glomerulopathies (84). The most associated genetic finding is the rearrangement of the CFH gene locus that leads to the formation of new CFHR fusion genes, which are then translated into new fusion proteins (FHR1-FHR1, FHR3-FHR3, FHR2-FHR2, FHR5-FHR-5, and FHR5-FHR2) that bind to the glycocalyx of the GBM and act as competitive inhibitors of factor H (85).

Assessment of the complement markers that demonstrate uncontrolled activation of the alternative complement pathway may be informative in establishing the diagnosis and monitoring of disease activity in C3 glomerulopathies (86). These include markers of (a) specific activation of the alternative complement pathway (low C3, normal C4, low CFB), (b) C3 turnover (low C3a and increased C3 degradation products), and (c) C5 turnover (low C5, high sC5b-9, and C5a). Most studies and societal guidelines recommend measuring these complements in the serum. While there is limited data to support urinary complement markers in measuring disease activity and treatment response, early studies are promising.

A case-control study by May et al. investigated the urinary complement biomarkers in 25 patients with biopsy-proven C3 glomerulopathy (11 of them were on eculizumab, a C5 inhibitor). It was demonstrated that C3 glomerulopathy is associated with elevated levels of urinary complement proteins (C3, C4, FB, properdin, and C5) and complement activation products (C3a, Ba, Bb, C5a, and soluble C5b-9). Of these, only urinary soluble C5b-9 (sC5b-9) levels correlated with the degree of proteinuria. The study also demonstrated that the urinary sC5b-9 levels normalized after treatment with eculizumab, highlighting its role in assessing treatment response. Interestingly, the urinary excretion of sC5b-9 did not correlate with the plasma levels of sC5b-9, confirming that local complement activation is associated with kidney disease activity in C3 glomerulopathy (87). These findings are similar to the preliminary findings of a clinical trial studying the effect of an unknown complement blocking agent on plasma and urinary Ba and sC5b-9 levels. This study showed that urinary Ba and sC5b-9 were significantly higher in patients with C3 glomerulopathy compared to healthy controls and normalized with drug treatment. This study also demonstrated that urinary levels of Ba and sC5b-9 showed a better correlation with both the presence of the disease and treatment response compared to plasma levels in C3 glomerulopathy (88).

These results indicate that urinary Ba and sC5b-9 reflect kidney complement activation and may be promising markers to assess local disease activity and treatment response in C3 glomerulopathy.

### 7.2 ANCA-associated vasculitis

The term ANCA-associated vasculitis refers to a group of pauci-immune small vessel vasculitis characterized by antibodies directed against proteins stored within neutrophilic granules, myeloperoxidase (MPO), and proteinase-3 (PR3). Historically, AAV was not thought to be a complement-mediated disease, given the absence of hypocomplementemia and limited complement deposition on kidney biopsies. However, recent animal studies have revealed that activation of the alternative complement pathway, specifically generation of anaphylatoxin C5a and C5a receptor (C5aR), plays a vital role in the pathogenesis of AAV (89–91). An amplification loop in which activated neutrophils release properdin promotes the alternative pathway and generates the anaphylatoxin C5a, which then binds to C5a receptors on neutrophils, leading to further neutrophil priming and activation, has been proposed. The crucial role of C5a is further supported by the fact that treatment with an anti-C5 antibody or C5aR antagonist is protective in murine models (91, 92). Recently, avacopan, a complement C5a receptor, has been approved to treat ANCA-associated vasculitis (93) and associated with improved renal recovery (94). Previous studies in kidney pathology have shown that the alternative pathway activation products could be detected in ANCA-associated glomerulonephritis (95–97). There is conflicting evidence regarding the significance of urinary complement activation products in assessing disease activity and treatment response in patients with ANCA-associated glomerulonephritis.

A study by Gou et al. investigated the products of alternative complement pathway activation in the urine of 27 patients with active ANCA-associated glomerulonephritis and compared it with patients in remission and healthy controls. The study demonstrated that markers of alternative complement pathway and terminal pathway activation, such as urinary Bb, C3a, C5a, and sC5b-9, were significantly higher in patients with active ANCA-associated glomerulonephritis as compared to healthy controls. There was no significant correlation between levels of complement products in the urine and plasma during active disease. In patients with ANCA-associated vasculitis, urinary Bb levels normalized in remission but urinary C3a, C5a, and sC5b-9 remained elevated (98). One study has also demonstrated elevated levels of urinary C1q and MBL in patients with ANCA-associated vasculitis compared to healthy controls, however, these levels were also significantly elevated in remission stage suggesting that activation of the classical and MBL pathway may be observed but is not pathogenic in AAV (98).

Urinary excretion of complement products has also been shown to correlate with disease activity in ANCA vasculitis. A study by Yuan et al. that included ~20 vasculitis patients demonstrated that the urinary levels of C5a were significantly higher in patients with AAV in the active phase than in patients with AAV in remission and normal controls (99). These findings were similar to the result of another cross-sectional study that demonstrated a significant difference in urinary C5a excretion between active and inactive diseases. In contrast, no such difference was observed in urinary levels of C5b and sC5b-9 (100). Another study by Almaani et al. compared the levels of urinary Ba and plasma Ba in 20 vasculitis patients with active renal flare, non-renal flare, and remission. This study demonstrated that urinary levels of Ba were higher in vasculitis patients with renal flare, normal in non-renal flare, and remained stable in remission. In this study, the urinary Ba levels showed a better correlation with active renal vasculitis compared to plasma Ba levels, suggesting its role as a surveillance biomarker of renal vasculitis (101).

Another study by Gou et al. demonstrated that urinary levels of Bb have a direct correlation with serum creatinine and the proportion of total crescents on renal biopsy, and an inverse correlation with the proportion of normal glomeruli on renal biopsy (98). Another study by Khalili et al. that included 83 patients with different immune-mediated glomerulopathies including AAV demonstrated a positive correlation between initial proteinuria and urinary sC5b-9 levels. In clinical remission, the urinary levels of sC5b-9 showed a greater reduction than the decline in proteinuria, suggesting an earlier and more precise variation in urinary sC5b-9 with disease activity (102).

These results indicate that urinary complement activation products, particularly markers of alternative complement pathway and terminal pathway activation, are useful in assessing disease activity and treatment response in patients with ANCA-associated glomerulonephritis.

### 7.3 Thrombotic microangiography

Thrombotic microangiopathy (TMA) is a clinical disorder characterized by thrombocytopenia, microangiopathic hemolytic anemia, and microvascular thrombosis resulting in systemic organ damage. Atypical HUS (aHUS) is a rare form of complement-mediated thrombotic microangiopathy that results from dysregulation of the alternative complement pathway in the solid phase. The alternative pathway dysregulation in aHUS may occur due to genetic alterations in complement factor H (most common), Factor I, Factor B, and C3, or autoantibodies against factor H. Like aHUS, complement-mediated TMA has also been described in patients with lupus nephritis and as a complication of hematopoietic stem cell transplant.

A study by Sertain et al. demonstrated that plasma Ba levels were significantly elevated in patients with transplantation Associated-TMA and showed an inverse correlation with eGFR (103). However, it should be noted that plasma Ba levels correlate strongly with GFR and may not necessarily represent complement activation (104). Another prospective study showed that subjects undergoing hematopoietic stem cell transplant who had elevated levels of sC5–9 at the time of transplant associated-TMA diagnosis had poor survival compared to subjects with normal C5b-9 (105). These findings suggest that dysregulation of the alternative complement pathway might be the pathogenic mechanism behind kidney injury in these patients. Another study by Fujiyama et al. demonstrated that serum levels of Ba were significantly elevated in patients who had TMA after kidney transplant (KT-TMA), suggesting its role as a candidate marker for KT-TMA (106).

Several studies in aHUS have identified the role of measuring serum complement proteins to assess disease activity. There is only one study that investigates the role of urine complement proteins in assessing disease activity and treatment response. A prospective trial by Cofiell et al. investigated the effect of eculizumab on biomarkers related to thrombotic microangiopathy in ~30 patients with aHUS. The study demonstrated that markers of terminal complement activation such as urinary C5a and sC5b-9 levels were significantly elevated (45-fold and 310-fold, respectively) in ~85% of patients with active disease. These patients demonstrated a rapid and sustained reduction in urinary C5a and sC5b-9 after eculizumab treatment (107).

A study by Mejia-Vilet et al. compared the plasma and urine complement activation products between patients with active lupus nephritis and those with acute TMA plus concomitant active lupus nephritis. The study demonstrated that urinary C3a, C5a, Ba, and C5bC9 were higher in patients with acute TMA plus concomitant active lupus nephritis and decreased with treatment (108).

These results indicate that alternative pathway dysregulation plays a central role in the pathogenesis of complement-medicated TMAs. Urine complements biomarkers have shown promising potential as non-invasive diagnostic and prognostic tools in various complement-mediated kidney diseases (summarized in **Table 2**). Urinary markers of terminal complement activation such as C5a and sC5b-9 can potentially be promising markers of disease activity and treatment response in aHUS. Similarly, urinary C3a, C5a, Ba, and C5bC9 may be potential markers for acute TMA with and without lupus nephritis.

**Table 2:** Urine complement biomarkers in various kidney diseases.

Disease	Urine complement proteins identified	Markers of disease activity
Immune-mediated kidney diseases		
**Lupus nephritis**	C3d, C4	C3d, C4
**IgA nephropathy**	C3a, C5a, C4d, MBL, C5b-9, fH, properdin	C4d (strongest), C3a, C5a, MBL, C5b-9, CFH, properdin
**Primary membranous nephropathy**	C5b-9	C5b-9
**C3 glomerulonephritis**	C3, C3a, C4, C5, C5a, Ba, Bb, C5b-9, CFB, properdin	C5b-9, Ba
**ANCA-associated vasculitis**	C1q, C3a, C5a, Bb, MBL, C5b-9	Bb, Ba, C5b-9
**Thrombotic microangiopathies (TMA)**		
**Atypical HUS**	C5a, C5b-9	C5a, C5b-9
**TMA in lupus nephritis**	C3a, C5a, Ba, C5b-9	C3a, C5a, Ba, C5b-9

## 8 The future of urinary complement biomarkers

Urine complements biomarkers have shown promising potential as non-invasive diagnostic and prognostic tools in various complement-mediated kidney diseases. Most of the published studies on complement mediated kidney diseases are cross-sectional or retrospective studies that have established a correlation of urine complement protein excretion with histological characteristics on kidney biopsy, traditional markers of disease activity, and markers of kidney damage (proteinuria, GFR etc.). However, only a few have attempted to investigate the temporal association and the role of urinary complement in predicting flares and treatment response in prospective studies.

Discovery of novel urinary complement protein measurement will be most useful if they overcome the limitation of the existing diagnostic and prognostic markers of these kidney diseases. Although the levels of urinary complement proteins have been shown to correlate with histological features on kidney biopsy, they are not a substitute for kidney biopsy in establishing the diagnosis of complement mediated kidney diseases since they are not specific to a particular disease. However, once the diagnosis is established, serial measurement of the urinary complement proteins can be useful in monitoring disease activity, prognostication, predicting flares, and assessing treatment response. Therefore, prospective studies that study the association between levels of urinary complement proteins with disease flares and treatment response are needed to validate the usefulness of these identified urinary complement biomarkers. Novel urinary complement markers will be most useful if they are able to differentiate active renal from non-renal disease and if changes in them precede the occurrence of disease flare and correlate with treatment response.

## 9 Complement specific therapies for complement mediated kidney diseases

Paralleled by an increasing understanding of the role of complement activation in immune-mediated and non-immune mediated kidney diseases, there is an increasing interest in the development of novel drugs targeting the complement system (109). While the clinical arsenal is limited at this time, there are many drugs in the pipeline that may soon expand the available options and therapeutic indications for complement specific therapies (110). (Summarized in **Table 3**). FDA-approved drugs, such as eculizumab and ravulizumab, which target C5, have been shown to be very effective in managing atypical HUS (111, 112). Their applications in other diseases such as C3GN is being investigated, albeit with mixed results. Additionally, the recent FDA approval of avacopan, a C5a receptor inhibitor for ANCA-associated vasculitis signifies a shift towards more targeted interventions that minimize side effects associated with broader immunosuppression (113). Additionally, investigational drugs targeting C5 such as Crovalimab (NCT04861259) and gefurulimab (NCT06208488) (C5 inhibitors), Cemdisiran (a siRNA targeting the C5 mRNA transcript- NCT03841448, NCT03999840) are in various stages of clinical trials in several glomerular diseases, including an atypical HUS, IgA nephropathy, lupus nephritis, membranous nephropathy and focal segmental glomerulosclerosis (NCT05314231).

**Table 3:** Complement therapeutics approved and under investigation for immune-mediated kidney disease.

Drug	Target	Approved for	Investigated in
**Eculizumab**	C5	aHUS (23738544)	• C3GN (NCT01221181) • Dense deposition disease. (NCT01221181)
**Ravulizumab**	C5	aHUS (30767127)	• IgAN (NCT06291376, NCT04564339) • LN (NCT04564339)
**Avacopan**	C5a receptor inhibitor	ANCA associated vasculitis (33596356)	• C3GN (NCT03301467)
**Crovalimab**	C5		• aHUS (NCT04861259)
**Gefurulimab**	C5		• Healthy adults (NCT06208488)
**Cemdisiran**	siRNA targeting the C5 mRNA transcript		• aHUS (NCT03303313, NCT03999840) • IgAN (NCT03841448)
**Pegcetacoplan**	C3		• TMA (NCT05148299) • C3GN and IC-MPGN (NCT04572854, NCT05809531) • C3GN and IgAN (NCT05067127, NCT04729062)
**ARO-C3**	C3		• Complement mediated kidney diseases (NCT05083364)
**IONIS-FB-LRx**	Factor B		• IgAN (NCT04014335)
**Iptacopan**	Factor B		• IC-MPGN (NCT05755386) • C3GN (NCT04817618)
**Danicopan**	Factor D		• C3 GN and IC-MPGN (NCT03369236, NCT03124368)
**Narsoplimab**	MASP-2 inhibitor		• IgAN (NNCT03608033)

Parallel developments are occurring with drugs targeting C3 and the lectin pathway, addressing the earlier stages of the complement cascade which are critical in many renal pathologies. Danicopan (NCT05162066), iptacopan (NCT04817618, NCT05755386) and Pegcetacoplan (NCT04729062) targeting Factor D, Factor B, and C3 respectively, have shown promise in PNH but are being trialed for diseases like C3 glomerulopathy and IgA nephropathy. The investigational C3 targeting drugs include novel agents like ARO-C3 (NCT05083364) and IONIS-FB-LRx (NCT04014335), which employ RNA interference and antisense technology to modulate complement activity at the genetic level.

Narsoplimab (NCT03608033), a novel, fully human monoclonal antibody against MASP-2 targets the MBL pathway and is currently being investigated in IgA nephropathy. These approaches could offer more refined control over complement-driven inflammation and injury in the kidneys, potentially leading to treatments that are both more effective and have fewer side effects. This ongoing shift towards precision medicine in the realm of complement therapeutics holds the promise of fundamentally altering the management of complement-mediated kidney diseases, offering hope for more targeted and sustainable interventions.

## 10 Conclusion

Complement dysregulation plays a central role in the pathogenesis and progression of many immune-mediated kidney diseases. The urinary excretion of complement peptides better correlates with complement deposition on kidney biopsies, the degree of damage on kidney biopsies, kidney disease activity, kidney damage (as evidenced by proteinuria and serum creatinine), and prognosis as compared to the conventional serum markers of disease activity. It has also been demonstrated that the urinary levels of some of these complement peptides decline with treatment and serve as better predictors of kidney recovery compared to conventional serum markers. Therefore, measuring and monitoring the dynamic changes in the complement peptides in the urine, rather than the absolute levels of proteins in serum samples, may better reflect the disease activity and pathogenic mechanisms. Urinary complement biomarkers, therefore, have a promising potential to serve as biomarkers of these kidney diseases and thus aid in guiding intervention. Many of these promising urinary biomarkers will need to be validated in larger studies.

## Author contributions

VK: Methodology, Writing – original draft, Writing – review & editing. MB: Writing – original draft, Writing – review & editing. JK: Conceptualization, Funding acquisition, Investigation, Methodology, Resources, Supervision, Writing – review & editing. SW: Conceptualization, Funding acquisition, Investigation, Methodology, Supervision, Writing – original draft, Writing – review & editing.
